# Antiapoptotic Gene Genotype and Allele Variations and the Risk of Lymphoma

**DOI:** 10.3390/cancers15041012

**Published:** 2023-02-05

**Authors:** Osama M. Al-Amer, Rashid Mir, Abdullah Hamadi, Mohammed I. Alasseiri, Malik A. Altayar, Waseem AlZamzami, Mamdoh Moawadh, Sael Alatawi, Hanan A. Niaz, Atif Abdulwahab A. Oyouni, Othman R. Alzahrani, Hanan E. Alatwi, Aishah E. Albalawi, Khalaf F. Alsharif, Ashraf Albrakati, Yousef M. Hawsawi

**Affiliations:** 1 Department of Medical Laboratory Technology, Faculty of Applied Medical Sciences, University of Tabuk, Tabuk 47713, Saudi Arabia; 2 Department of Biology, Faculty of Sciences, University of Tabuk, Tabuk 71491, Saudi Arabia; 3 Genome and Biotechnology Unit, Faculty of Sciences, University of Tabuk, Tabuk 71491, Saudi Arabia; 4 Department of Clinical Laboratory Sciences, College of Applied Medical Sciences, Taif University, Taif 21944, Saudi Arabia; 5 Department of Human Anatomy, College of Medicine, Taif University, Taif 21944, Saudi Arabia; 6 Research Center, King Faisal Specialist Hospital and Research Center, MBC-J04, P.O. Box 40047, Jeddah 21499, Saudi Arabia

**Keywords:** lymphoma, whole exome sequencing, antiapoptotic genes, *BCL2*, *MCL1*, *BIRC5*

## Abstract

**Simple Summary:**

The results of prior studies on the genotypes and allele variations of the antiapoptotic gene and the risk of lymphoma are unclear. To address this gap, this case–control study included 205 Saudi patients, 100 of whom had lymphoma, and 105 who were healthy controls. Antiapoptotic genes, including B-cell lymphoma-2 (BCL2-938 C > A), *MCL1* (rs9803935 T > G), and *survivin* (BIRC5-rs17882312 G > C and BIRC5-rs9904341 G > C) were identified using the tetra amplification refractory mutation polymerase chain reaction (PCR). *BIRC5-C*, *MCL1-G*, and *BIRC5-G* alleles were identified using allele-specific PCR. In addition to *BCL-2-A* mutations, lymphoma patients are more likely to have antiapoptotic gene genotypes and allele variations than healthy volunteers. The results could help classify and identify those at risk of lymphoma in the future.

**Abstract:**

Background: The findings of earlier investigations of antiapoptotic gene genotypes and allele variants on lymphoma risk are ambiguous. This study aimed to examine the relationship between the mutation in the antiapoptotic genes and lymphoma risk among Saudi patients. Methods: This case–control study included 205 patients, 100 of whom had lymphoma (cases) and 105 who were healthy volunteers (controls). We used tetra amplification refractory mutation polymerase chain reaction (PCR) to identify antiapoptotic genes such as B-cell lymphoma-2 (BCL2-938 C > A), MCL1-rs9803935 T > G, and *survivin* (BIRC5-rs17882312 G > C and BIRC5-rs9904341 G > C). Allelic-specific PCR was used to identify alleles such as *BIRC5-C*, *MCL1-G*, and *BIRC5-G*. Results: The dominant inheritance model among cases showed that mutations in all four antiapoptotic genes were more likely to be associated with the risk of lymphoma by the odds of 2.0-, 1.98-, 3.90-, and 3.29-fold, respectively, compared to controls. Apart from the *BCL-2-A* allele, all three specified alleles were more likely to be associated with lymphoma by the odds of 2.04-, 1.65-, and 2.11-fold, respectively. Conclusion: Unlike healthy individuals, lymphoma patients are more likely to have antiapoptotic gene genotypes and allele variants, apart from *BCL-2-A* alterations. In the future, these findings could be used to classify and identify patients at risk of lymphoma.

## 1. Introduction

Lymphoma is caused by the clonal expansion of lymphocytes at various phases of cell maturation [1,2]. Approximately 72% of lymphoma patients are still alive after five years, and this percentage is rising due to improvements in scientific research and development [1]. However, there are two main types of lymphomas: Hodgkin lymphoma (HL) and non-Hodgkin lymphoma (NHL). NHL accounts for 90% of cases, and HL accounts for 10% of cases [3]. The B-cell, T-cell, and natural killer (NK) cell types are further categorized as NHL [4]. 

Hyperproliferation increases the possibility of genetic harm. Genetic anomalies and transcription factors active during several cell cycles promote lymphomagenesis [5,6]. Despite the variability in NHL, lymphomagenesis may be influenced by downstream cascades of crucial regulatory mechanisms such as apoptotic pathways [7,8]. The antiapoptotic *BCL-2* family gene of the *MCL1* apoptosis regulator was identified as an early response gene [9,10]. *MCL1* is necessary for both healthy and cancerous B cells to survive and become immortal [11]. A full-length antiapoptotic (full-length isoform) or pro-apoptotic (short isoform) gene is produced as a result of transcriptional and posttranscriptional control of *MCL1* expression [12]. *MCL1* gene amplifications were found to be frequent chromosomal gains that aided in survival in a study of more than 3000 samples encompassing 26 different tumor types [13]. Both solid tumors and hematopoietic system neoplasms frequently exhibit *MCL1* abnormalities. Moreover, it has been demonstrated that B-cell lymphoma, primarily of the aggressive kind, develops in *MCL1* transgenic mice [14]. According to Nizar et al. [15], increased levels of the antiapoptotic protein *MCL1*, which slows programmed cell death, have been linked to decreased chemotherapy sensitivity and worse prognoses for cancer patients. 

The *BCL2* apoptosis regulator gene has two promoters, each of which has a specific function [16]. The P1 promoter stimulates transcription, whereas the P2 promoter acts as a negative regulator. As a result of the increased activity of the P2 promoter due to cis alterations, *BCL2* expression is decreased, as has been shown [17]. A mutation is present in the 938 C > A operational region of the P2 promoter [18]. The C and A alleles of this mutation affect promoter activity differently, depending on how they bind to transcription factors. Lower *BCL2* expression results from a change in transcription factor binding, higher inhibitory promoter P2 activity, and altered transcription factor binding. Additionally, the association between genetic variants containing the 938 C > A allele and cancer progression has been investigated by examining different cancers in diverse populations. In chronic lymphocytic leukemia patients, B cells demonstrated that the 938 C allele is significantly correlated with enhanced P2 promoter activity, which has the opposite effect of reducing *BCL2* gene expression. The single nucleotide polymorphisms (SNPs) of the BLC2 gene have been linked to various malignancies, including chronic lymphocytic leukemia [19,20,21,22,23,24,25]. Park et al. used region sequencing on 24 Korean female DNA samples to discover the 938 C > A SNP in the P2 promoter of the *BCL2* gene [26]. However, the 938 C > A allelic variants and *BCL2* gene expression have not been linked by specific research [27].

The human *survivin* gene was found to play a critical role in the emergence of cancer by accelerating the accumulation of genetic changes, obstructing intrinsic and extrinsic apoptotic pathways, and enhancing sensitivity to therapy [28]. In contrast to healthy adult tissues, this gene is infrequently expressed in malignant tissues [29]. As a result, owing to its function in blocking apoptosis, *survivin* mutation may change the expression of *survivin*, which is necessary for the emergence and spread of cancer [30,31]. The *survivin* gene contains several mutations. Several of these include the missense variants rs1042489 in the 3’ untranslated region; rs8073069, rs17878467, and rs9904341 located in the promoter; and the missense variant rs2071214 in exon 5, for which the relationship between *survivin* mutations and cancer susceptibility in various populations has been evaluated in numerous studies [32,33,34,35,36]. *Survivin* overexpression has been associated with poor prognosis in several cancers, including gliomas, renal cell carcinoma, esophageal cancer, breast cancer, gastric cancer, ovarian cancer, laryngeal cancer, and colorectal cancer [37,38,39,40,41,42,43,44]. The findings of this study are inconsistent, although some assert the *survivin* gene indicates lymphoma patients would have a worse prognosis [45,46,47]. 

The results of the studies mentioned above regarding the genotypes, allele variants, and lymphoma risk of *BCL2*-938 (rs2279115 C > A), *MCL1* (rs9803935 T > G), *survivin*-141 (rs17882312 G > C), and *survivin*-31 (rs9904341 G > C) are inconsistent. Therefore, this study aimed to investigate the association between mutations in antiapoptotic genes and the risk of lymphoma in Saudi patients. According to the study’s hypothesis, the risk of lymphoma in this patient population is significantly influenced by mutations in antiapoptotic gene genotypes and alleles.

## 2. Materials and Methods

### 2.1. Sample Collection

Patients with histologically proven aggressive lymphoma were included in the trial if they had clinically confirmed lymphoma. We collected histological slides from the hospital pathology department, including those with H&E staining, immunohistochemical staining, and other specialized research. The pathological diagnosis of lymphoma is aided by the histological patterns of the disease, which are discernible in the core sample tissue. Samples were collected after receiving a letter of consent from the relevant institutional ethics committee.

Participants aged between 20 and 70 years who visited a hospital for a routine checkup were used to create a healthy control group. A population from the same geographic area was used to select controls. Healthcare professionals performed a standard routine medical examination (CBC, KFT, LFT, etc.) and noted any illnesses discovered. They were considered normal if they appeared healthy and had no known history of a severe disease or other chronic conditions. Socio-demographic details, including age, sex, and lifestyle, were recorded using a standard questionnaire. Peripheral blood samples were used in the cohort of healthy volunteers.

### 2.2. Genomic DNA Extraction

The histological patterns of lymphoma that can be seen in core biopsy tissue, mainly archived bone marrow (the spongy part inside the bone where blood cells are made) tissue, were used to make a pathological diagnosis of lymphoma. The diameter of the core tissue was calculated based on the inner diameter of the cutting needles. Tissue volume was computed using the formula: volume = (diameter/2)2 length 3.14. The comprehensive methodology for this straightforward technique is presented in Supplementary Table S1. According to the manufacturer’s instructions, DNA was extracted using a deparaffinization solution (Cat. No./ID: 19093) and the QIAamp DNA FFPE tissue kit from Qiagen (Cat. No./ID: 56404, Washington, USA). The DNA was then dissolved in nuclease-free water and kept at 4 °C until use. The isolated DNA was dissolved in water devoid of nucleases and kept at 4 °C until needed. We evaluated the quality of the extracted DNA by putting the sample through a 1% agarose gel. A spectrophotometer, or NanoDropTM, was used to measure absorbance at 260 nm and 280 nm to determine the amount of the extracted DNA (Thermo Scientific, USA, Waltham, MA, USA).

### 2.3. Genotyping of BCL2, MCL1, and Survivin Genes

Amplification refractory mutation system PCR and allele-specific PCR were used to determine the genotypes of *BCL2-938* (rs2279115 C > A), *MCL1* (rs 9803935 T > G), *survivin*-141 (rs 17882312 G > C), and *survivin*-31 (rs9904341 G > C) mutations. Primers for the genotypes of *BCL2* (rs 2279115 C > A), *MCL1* (rs 9803935 T > G), *survivin*-141 (rs 17882312 G > C), and *survivin*-31 (rs 9904341 G > C) are shown in Supplementary Table S2.

*BCL2-938* (rs 2279115 C > A): A band of 300 bp was produced using the *BCL2* gene’s primers FO and RO to regulate DNA quality and quantity. A band of 121 base pairs (bp) was produced by the wild-type allele (C allele) amplified by primers Fwt and RO, and a band of 220 bp was produced by the mutant allele using primers FO and Rmt (A allele).

*MCL1-rs 9803935*: After Fo and R0 amplified the *MCL1* outer region, a 276 bp band was created that serves as a DNA purity indicator. Using the primers F0 and RI, the A allele generated a band of 144 base pairs, and the G allele generated a band of 190 base pairs.

*Survivin*-141 (rs 17882312 C > G): Primers F1 and R of the survivin-141 gene produced a band of 360 bp from the C allele. A band of 360 bp was produced by the amplification of the polymorphic G allele using primers F2 and R.

*Survivin*-31 (rs 9904341 G > C): Primers F1 and R for the *survivin*-31 gene created a band of 357 bp from the G allele. A band of 360 bp was produced by amplifying a polymorphic allele (C allele) using primers F2 and R.

### 2.4. Sequencing

We carried out whole exome sequencing on 20 clinically annotated cases to better understand lymphoma patients’ mutational and molecular landscape. The results are shown in Supplementary Table S3. The reported case on the personalized gene panel served as the basis for using the Illumina Novaseq 6000 platform for next-generation sequencing to identify *BCL2* gene alterations. 

We used the FFPE DNA isolation kit (TANbead, Cat. No. M61PS46, Taoyuan City, Taiwan) to isolate DNA from FFPE Curls. DNA was quantified using Qubit DNA Assay BR (Invitrogen, Cat. No. Q32853, Waltham, MA, USA). DNA integrity was checked using Genomic DNA Screen Tape (Agilent, Cat. No. 5067-5365, Waldbronn, Germany). All the samples that passed the DNA QC were taken for Targeted Library preparation.

Targeted exome capture was performed using a custom Twist panel. Biotinylated oligonucleotide capture probes (Twist Biosciences, South San Francisco, CA, USA), also called baits, were used to enrich by hybridization. The workflow involves enzymatic shearing of DNA, repairing ends, adenylation of 3’ ends, followed by adapter ligation using Twist EF Library Preparation kit (Cat. No. 101058). At each step, the products were purified using Twist Binding and Purification beads (Cat. No. 100984). We added the Illumina adapters onto the ends of DNA fragments to generate paired-end libraries. The resulting adaptor-ligated libraries were purified, qualified, and hybridized with target-specific biotinylated capture probes. After hybridization, the targeted molecules were captured using streptavidin magnetic beads. The resulting enriched DNA libraries were multiplexed by adding index tags by amplification, followed by purification. The captured library was then assessed for fragment size distribution on a tape station using D1000 ScreenTape (Agilent, Cat. No. 5067-5582).

Prepared libraries were quantified using the Qubit High Sensitivity reagent (Invitrogen, Cat. No. Q32851). The libraries were diluted appropriately and pooled for cluster amplification and sequencing on NovaSeq 6000 to generate 2 × 150 bp reads. We processed the sequenced data to create FASTQ files and uploaded them on the sFTP server for download and secondary analysis.

### 2.5. Statistical Analysis

We determined the change from Hardy–Weinberg equilibrium using the chi-square (2) goodness-of-fit test and disequilibrium. Student’s two-sample *t*-test or one-way analysis of variance (ANOVA) for continuous variables and the chi-squared test for categorical variables were used to determine the genotype and allele differences in BCL2-938 (rs 2279115 C > A), *MCL1* (rs9803935 G > T), *survivin*-141 (rs 17882312 G > C), and *survivin*-31 (rs 9904341 G > C). We calculated the relationships between the *BCL2-938* (rs2279115 C > A), *MCL1* (rs 9803935 G > T), *survivin*-141 (rs 17882312 G > C), and *survivin*-31 (rs 9904341 G > C) genotypes and leukemia susceptibility using odds ratios (ORs), risk ratios (RRs), and risk differences (RDs) with 95% confidence intervals (CIs). To calculate the univariate and multivariate analyses, we defined a statistically significant difference as having a *p*-value of 0.05 or lower. We used software version 20.02 of MedCalc (SPSS Inc., Ostend, Belgium).

## 3. Results

### 3.1. Characteristics of Cases

The demographic characteristics of the participants are listed in Table 1. Of the participants, 100 were clinically confirmed lymphoma cases, and 105 were healthy volunteers. The patients with lymphoma were predominantly male (63%) and under 45 years of age (56%). All patients had healthy weights (24.4 kgm^2^). About 90% of the patients had non-Hodgkin lymphoma, and 55% had it in an advanced stage. Most healthy volunteers were males (60%) aged > 45 years (61.9%) and had healthy weights (24.7 kg/m^2^).

The association of genotypes of BCL2-938 (rs 2279115 C > A), *MCL1* (rs 9803935 G > T), *survivin*-141 (rs 17882312 G > C), and *survivin*-31 (rs 9904341 G > C) concerning patient clinical characteristics is depicted in Table 2. Male sex was more significant in all three genotypes except Survivin-rs 9904341 G > C gene. However, the age > 45 years was substantial for all four genotypes. All four genotypes were significantly associated with early-stage lymphoma, especially non-Hodgkin lymphoma.

### 3.2. Distribution of Genotype Mutations between Cases and Controls

The distribution of genotype mutations between cases and controls is shown in Table 3. The *BCL2-938 C > A* gene showed a statistically significant (*p* < 0.033) variance between cases and controls. The frequency of the A allele was also higher in the patients (0.34) than in the controls (0.26). *BCL2-938 C > A* genotype frequencies in patients were CC (37%), CA (58%), and AA (5%), whereas those in controls were CC (54.28%), CA (40%), and AA (5.71%).

The *MCL1-rs 9803935 T > G* gene mutation significantly differed between patients and controls (*p* < 0.0001). Additionally, compared to the controls, cases had a greater prevalence of the G allele (0.37 vs. 0.27). The most prevalent genotypes in the patients were TT (36%), TG (54%), and GG (10%), whereas the most prevalent genotypes in the controls were TT (52.7%), TG (40.9%), and GG (6.36%).

A statistically significant mutation in the *survivin-rs 17888231 G > C* gene was identified between the cases and controls (*p* = 0.003). Cases and controls had genotype frequencies of CC (31%), GC (65%), and GG (4%), respectively. Additionally, cases had a higher frequency of the G allele than the controls (0.36 vs. 0.21).

We identified a statistically significant difference in the *survivin-rs 9904341 G > C* gene between patients and controls (*p* < 0.0001). Additionally, healthy controls had a greater C allele frequency than the patients (0.42 vs. 0.38). The frequencies of G > C genotypes in patients and controls were GG (29%), GC (66%), and CC (5%), respectively; for controls, they were CA (40.9%) and AA (1.63%).

### 3.3. Association between BCL2-938 C > A Genotypes and Lymphoma Risk

The findings showed that the BCL2-CA genotype was significantly related to higher risks of lymphoma by 2.12 (95% CI = 1.19–3.77) among patients than the controls. In other words, the BCL2-CA genotype increased the risk of lymphoma by 1.44 times (95% CI = 1.089–1.91) compared to the controls. In the case of the dominant inheritance model, the BCL2-CC and BCL2-(CA + AA) genotypes were significantly associated with a double (OR = 2.02, 95% CI = 1.156–3.53) odds and a 1.40-fold (95% CI = 1.07–1.83) increase in the risk of lymphoma among cases compared with controls. BCL2-CA and BCL2-CC + AA genotypes were associated with a lower risk of lymphoma (OR = 0.48, 95% CI = 0.277–8.04; RR = 0.70, 95% CI = 0.53–0.92) (Table 4). 

### 3.4. Association between MCL1-rs9803935 T > G Genotypes and Lymphoma Risk

In the codominant inheritance model, it was found that the variation in the *MCL1-TG* genotype was substantially related to the odds of lymphoma by 1.93 (95% CI = 1.08–3.43) and 1.35 (1.03–1.77) times more in cases than controls. In the dominant inheritance model, the *MCL1-TT* and *MCL1 (TG + GG)* genotypes were highly related, with odds of 1.98 (95% CI = 1.09–2.51) and 1.28 (1.03–1.61) times the increase in lymphoma risk, respectively. The risk of lymphoma was 1.65-fold (95% CI = 1.09–2.51) with the *MCL1-G* allele and 1.28 times (95% CI = 1.03–1.61) higher among cases than in controls (Table 5).

### 3.5. Association between Survivin-rs 17882312 G > C Genotypes and Lymphoma Risk

In the codominant inheritance model, the *BIRC5-GC* genotype was significantly associated with an increased risk of lymphoma by 1.64-fold (95% CI: 1.26–2.14) and 2.98 times (95% CI: 1.68–5.28) in cases compared to controls. According to the dominant inheritance model, the *BIRC5-GG* and *BIRC5 (GC + CC)* genotypes were linked to higher odds and an increased risk of lymphoma in cases compared to controls of 3.09 (95% CI = 1.754–5.46) and 1.68 (95% CI = 1.29–2.19), respectively. Similarly, the *BIRC5-G* allele was substantially linked to the odds of an increased risk of lymphoma by 2.11 (95% CI = 1.37–3.25) and 1.47 (95% CI = 1.15–1.87, respectively). The *BIRC5-GC* and *BIRC5-GG + CC* genotypes were significantly related to lower odds of lymphoma risk in the dominant inheritance model (OR = 0.37, 95% CI = 0.21–0.65), and the chance was significantly lower in cases compared to controls by 0.62 times (95% CI = 0.48–0.82) (Table 6).

### 3.6. Association between Survivin-rs 9904341 G > C Genotypes and Lymphoma Risk

In the codominant inheritance model, the *BIRC5-GC* genotype was significantly related to a 3.18-fold (95% CI: 1.80–5.62) and 1.64 times (95% CI: 1.28–2.09) increase in cases compared to controls. Similarly, the *BIRC5–AA* genotype was strongly associated with lymphoma risk, with odds of 6.0-fold (95% CI = 1.10–32.9) and an increased risk of 2.47 times (95% CI = 0.76–8.03) in cases compared to controls. According to the dominant inheritance model, the *survivin-GG* and *survivin (GC + CC)* genotypes were linked to higher odds and an increased risk of lymphoma in cases compared to controls of 3.29 (95% CI = 1.87–5.77) and 1.67 (95% CI = 1.31–2.13), respectively. Similarly, the *BIRC5-C* allele was substantially linked to the odds of an increased risk of lymphoma by 2.04 (95% CI = 1.35–3.00) and 1.30 (95% CI = 1.03–1.63), respectively. The *survivin*-*GC* and *BIRC5-GG + CC* genotypes were significantly related to lower odds of lymphoma risk in the dominant inheritance model (OR = 0.35, 95% CI = 0.20–0.61), and the chance was significantly lower in cases than in controls by 0.88 times (95% CI = 0.64–1.23) (Table 7).

## 4. Discussion

The *MCL1-rs9803935 T > G*, *Survivin-rs17882312 G > C*, *BIRC5-rs17882312 C > G*, and *Survivin-rs9904341 G > C* genotypes and alleles were found to be substantially related to the risk of lymphoma in patients compared to controls. The risk of lymphoma was higher with the *MCL1-G* allele, and a more significant increase was observed in patients than in controls. According to the dominant inheritance model, *BIRC5-GC* genotypes were associated with higher odds and an increased risk of developing lymphoma. The *BIRC5-GC* genotype was significantly associated with an increased risk of lymphoma in patients compared to controls. The *survivin-GG* and *survivin (GC + CC)* genotypes were associated with a higher risk.

Furthermore, the results exhibited that all three genotypes except *Survivin* (rs9904341) G > C were more significant in males. Age >45 years was important for all four genotypes. All four genotypes were significantly associated with NHL in its early stages. One explanation could be that the average risk of lymphoma—notable NHL—is more frequently higher in men than in women based on biological sex [48]. However, the mechanisms behind these variations are still unknown [49]. Based on data from Cancer Research UK, we chose a 45-year-old cutoff point because age-specific incidence rates increase steadily from about 45 to 49, then more dramatically from about 55 to 59. The age groups of 80–84 for females and 85–89 for males have the highest rates [50].

The functional BCL2-938 C > A promoter mutation has been demonstrated to affect the balance between malignant hematolymphoid cell survival and apoptosis [42]. According to several studies, *BCL2* is significantly overexpressed in most malignancies [51,52]. High *BCL2* expression has been reported in hematological malignancies and diffuse a large B-cell lymphoma, and the A allele is linked to greater *BCL2* expression [2]. Between lymphoma patients and controls, there was statistically significant variance in the *BCL2-938 C > A* gene. A higher allele frequency was found in lymphoma patients than in healthy controls. Contrary to controls, where genotype CC was more prevalent (54.28%), lymphoma patients were more likely to have the CA genotype of *BCL2-938 C > A* (58%), according to research [51]. In multivariate analysis, the codominant model’s *BCL2-CA* genotype was highly linked to an increased susceptibility to lymphoma. The dominant inheritance model revealed a significant correlation between the *BCL2-CC* and *BCL2-(CA + AA)* genotypes. However, the BCL2 (CC + CA) and BCL2 (AA) genotypes showed no correlation under the recessive inheritance paradigm. In an allelic comparison, the *BCL2-A* allele was not linked to lymphoma susceptibility; however, in an overdominant inheritance model, there is a reduced effect (protective genotype) between the *BCL2-CA* and *BCL2-CC + AA* genotypes.

Even though the relationship between *BCL2* mutations and the risk of lymphoma has previously remained unclear, a previous study assessed the frequency and type of *BCL2* mutations in two independent cohorts of grade 1 and 2 lymphomas, as well as the relationship between *BCL2* mutations, transformation risk, and survival [53]. The findings revealed that *BCL2* mutations are linked to increased activation-induced cytidine deaminase production, altered antiapoptotic *BCL2* function, and a higher probability of lymphoma transformation and death.

Two of the eight members of the human inhibitor of apoptosis protein (IAP) family are *BIRC5* (survivin) and *BIRC3* (cellular IAP2) [34]. The high expression of *BIRC5*, one of the top-scoring genes and one of the four with the best predictive value, was significantly associated with a worse patient survival rate [35,36]. The frequency of the C allele was higher in healthy controls than in lymphoma patients, making the *BIRC5-rs9904341 G > C* gene variation between lymphoma patients and controls statistically significant. The *G > C* genotype of *BIRC5-rs9904341* was most prevalent in lymphoma patients (66%), whereas the *GG* genotype was more prevalent in controls (57%) [27]. 

According to the study’s findings, the *BIRC5-GC* genotype is strongly linked to a patient’s vulnerability to developing lymphoma, and the *BIRC5-AA* genotype is also strongly linked to lymphoma susceptibility. According to the dominant inheritance model, there is a substantial correlation between the *BIRC5-GG* and *BIRC5 (GC + CC)* genotypes, which increases the vulnerability of lymphoma patients. The *BIRC5 (GG + GC)* and *BIRC5 (CC)* genotypes do not show any connection under the recessive inheritance paradigm. When allelic comparisons were made, the *BIRC5-C* allele was significantly related to lymphoma susceptibility. The *BIRC5-GC* and *BIRC5-GG + CC* genotypes exhibit a diminished effect (a protective genotype) under the recessive inheritance paradigm, as shown previously [29,31].

The gene variant seen between lymphoma patients and controls for *MCL-1rs 9803935 T > G* was statistically significant. Additionally, lymphoma patients had a higher frequency of the G allele than healthy controls [10]. In lymphoma patients, TG genotypes were more common (54%), while in controls, the TT genotype was more common (52.7%). The *MCL1-TG* genotype was significantly related to higher lymphoma patient susceptibility in the codominant model, whereas the *MCL1-GG* genotype was not. In the case of the dominant inheritance model, a strong association of *MCL1-TT* vs. *MCL1-(TG + GG)* genotypes was observed, which leads to increased lymphoma patient susceptibility. In the case of the recessive inheritance model, no association was observed between the *MCL1-(TT + TG)* and *MCL1-GG* genotypes. In allelic comparison, the *MCL1-G* allele was strongly associated with lymphoma susceptibility, while in the dominant inheritance model, no association was observed between the *MCL1-TG* and *MCL1-TT + GG* genotypes.

Survivin is an inhibitor of apoptosis family members that inhibit caspases and delay cell death in high concentrations in most malignancies. A worse clinical outcome is also associated with survivin [3]. Given the high frequency of GC genotypes in lymphoma patients—at 65%—the *survivin-rs17882322, G > C* gene variation seen between lymphoma patients and controls was statistically significant. The CC genotype was more prevalent (58.18%) among controls. It was also discovered that lymphoma patients had a higher frequency of the G allele than healthy controls. The codominant inheritance model’s multivariate analysis revealed a robust association between the *survivin* genotype and elevated lymphoma patient susceptibility. On the other hand, the *survivin* CC genotype was not linked to lymphoma susceptibility.

A total of 115 SNPs were found to be related to circulating total bilirubin in genome-wide association studies conducted in the UK Biobank [53,54]. The other 114 SNPs (non-UGT1A1 SNPs) accounted for 3.1% of the phenotypic variance in circulating bilirubin levels. At the same time, one SNP (rs6431625) in the promoter region of the uridine-diphosphoglucuronate glucuronosyltransferase 1A1 (UGT1A1) gene explained 16.9% of the phenotypic variance. A one-standard-deviation rise in circulating bilirubin (4.4 mol/L) predicted by non-UGT1A1 SNPs was inversely linked with a 0.64-fold risk of Hodgkin lymphoma (95% CI 0.42–0.99, *p* = 0.04) after putative pleiotropic SNPs were eliminated.

## 5. Conclusions

A higher risk of lymphoma, notable NHL, was shown to be related to variations in the antiapoptotic genes *BCL2-938 C > A, MCL1-rs9803935 T > G, survivin-rs17882231 G > C, and BIRC5-rs9904341 G > C,* particularly among men aged more than 45 years. The C allele in *BIRC5*, G allele in *MCL1*, and the G allele in *BIRC5* were all substantially associated with an elevated risk of lymphoma, with the exception of the BCL-2-A allele. Forty-nine variations identified using whole exome sequencing demonstrated a mutational burden on the *BCL2* gene’s role in lymphoma genesis. SNPs for each gene were associated with disease risk; however, in several instances, the SNPs for all three genes overlapped. Further investigation is required if combinations of two or more SNPs exhibit a more significant association with disease risk. Future identification and classification of patients at risk of lymphoma may benefit from these findings. Future longitudinal experiments using larger sample sizes and diverse ethnic communities are advised to validate these findings.

## Figures and Tables

**Table 1:** Characteristics of study participants.

Characteristic	Cases *n* = 100 (48.8%)	Controls *n* = 105 (51.2%)
**Age threshold, *n* (%)**		
Age >45	44 (44)	65 (61.9)
Age <45	56 (56)	40 (38.1)
**Gender, *n* (%)**		
Males	63 (63)	63 (60)
Females	37 (37)	42 (40)
**^π^** **BMI kg/m^2^**	24.40 ± 2.60	24.70 ± 2.60
**^π^** **FPG mmol/L**	-	4.70 ± 0.79
**^π^** **Free insulin mU/ml**	-	7.70 ± 2.70
**^π^** **HbA1c mmol/mol**	-	3.90 ± 0.48
**^π^** **Triglycerides mmol/L**	-	1.50 ± 0.59
**^π^** **Cholesterol mmol/L**	-	1.50 ± 0.63
**^π^** **LDL mmol/L**	-	1.85 ± 0.60
**^π^** **HDL mmol/L**	-	1.60 ± 0.70
**Lymphoma subtype**	-	-
**Diffuse large B-cell lymphoma**	45 (45)	-
**Follicular Lymphoma**	10 (10)	-
**Burkitt’s Lymphoma NHL**	10 (10)	-
**T-CELL NHL**	15 (15)	-
**MALT Lymphoma**	10 (10)	
**Hodgkin Lymphoma**	10 (10)	-

Abbreviations: BMI, body mass index; FPG, fasting plasma glucose; HbA1c, hemoglobin A1c; LDL, low-density lipoprotein; HDL, high-density lipoprotein. ^π^ Values are presented as mean ± standard deviation and (interquartile range).

**Table 2:** Demographic features of Lymphoma patients according to genotypes.

*BCL2-938 C > A*	*n*	%	CC	CA	AA	*df*	*X* ^2^	*p*-Value
**Sex**								
Males	63	63%	14	45	04	2	15.96	0.0003
Females	37	37%	23	13	01			
**Age group in years**								
Age >45	44	44%	10	30	4	2	8.36	0.015
Age <45	56	56%	27	28	1			
**Stage of lymphoma**								
Early stage	45	45%	10	32	3	2	7.71	0.02
Advanced stage	55	55%	27	26	2			
**Type of lymphoma**								
Non-Hodgkin Lymphoma	90	90%	32	56	2	2	17.16	0.0002
Hodgkin Lymphoma	10	10%	5	2	3			
**Bone marrow involvement**								
Yes	32	32%	10	20	2	2	0.73	0.69
No	68	68%	27	38	3			
***MCL1- rs9803935 T > G***	***n***	**%**	**TT**	**GT**	**TT**	***df***	***X* ^2^**	***p*** **-Value**
**Sex**								
Males	63	63%	15	44	06	2	12.13	0.002
Females	37	37%	21	12	04			
**Age group in years**								
Age >45	44	44%	22	16	6	2	9.84	0.007
Age <45	56	56%	14	38	4			
**Stage of lymphoma**								
Early stage	45	45%	21	18	6	2	6.36	0.039
Advanced stage	55	55%	15	36	04			
**Type of lymphoma**								
Non-Hodgkin Lymphoma	90	90%	30	50	8	2	2.43	0.29
Hodgkin Lymphoma	10	10%	06	04	02			
**Bone marrow involvement**			36	54	10			
Yes	32	32%	10	18	4	2	0.63	0.72
No	68	68%	26	36	6			
***Survivin-rs17882312 G > C***	***n***	**%**	**TT**	**GT**	**TT**	***df***	***X* ^2^**	***p*** **-Value**
**Sex**								0.0001
Males	63	63%	10	50	03			
Females	37	37%	21	15	01			
**Age group in years**								
Age >45	44	44%	8	33	3	2	6.93	0.031
Age <45	56	56%	23	32	1			
**Stage of lymphoma**								
Early stage	45	45%	10	31	04	2	7.11	0.028
Advanced stage	55	55%	21	34	0			
**Type of lymphoma**								
Non-Hodgkin Lymphoma	90	90%	27	60	03	2	1.67	0.12
Hodgkin Lymphoma	10	10%	04	05	01			
**Bone marrow involvement**								
Yes	32	32%	14	15	03	2	8.25	0.016
No	68	68%	17	50	01			
***Survivin-rs9904341 G > C***	***n***	**%**	**TT**	**GT**	**TT**	***df***	***X* ^2^**	***p*** **-Value**
**Sex**								
Males	63	63%	14	46	03	2	3.99	0.13
Females	37	37%	15	20	2			
**Age group in years**								
Age >45	44	44%	07	35	02	2	6.86	0.032
Age <45	56	56%	22	31	03			
**Stage of lymphoma**								
Early stage	45	45%	5	29	01	2	15.29	0.0004
Advanced stage	55	55%	24	27	04			
**Type of lymphoma**								
Non-Hodgkin Lymphoma	90	90%	27	59	04	2	0.89	0.64
Hodgkin Lymphoma	10	10%	2	7	1			
**Bone marrow involvement**								
Yes	32	32%	10	20	2	2	0.32	0.85
No	68	68%	19	46	03			

**Table 3:** Distribution of genes’ variation between cases and controls.

Groups by Genes	*n*				*Df*	*X* ^2^			*p*
*BCL2-938 C > A*		**CC**	**CA**	**AA**			**G**	**A**	
Cases	100	37 (37%)	58 (58%)	5 (5%)	2	6.79	0.64	0.34	0.033
Controls	105	57 (54.28%)	42 (40%)	6 (5.71%)			0.74	0.26	
*MCL1-rs9803935 T > G*		**TT**	**GT**	**GG**			**T**	**G**	
Cases	100	36 (36%)	54 (54%)	10 (10%)	2	6.03	0.63	0.37	0.049
Controls	105	58 (52.7%)	45 (40.9%)	07 (6.36%)			0.73	0.27	
*Survivin-rs17882312 G > C*		**CC**	**GC**	**GG**			**C**	**G**	
Cases	100	31 (31%)	65 (65%)	04 (4%)	2	14.4	0.64	0.36	0.003
Controls	105	64 (58.1%)	45 (40.9%)	1 (0.90%)			0.79	0.21	
*Survivin-rs9904341 G > C*		**GG**	**GC**	**CC**			**G**	**C**	
Cases	100	29 (29%)	66 (66%)	05 (5%)	2	18.4	0.62	0.38	0.001
Controls	105	70 (57%)	50 (40.9%)	02 (1.63%)			0.58	0.42	

**Table 4:** Association of *BCL2-938 C > A* gene variation in cases and controls.

Genotypes	Control *n* = 105	Cases *n* = 100	OR (95% CI)	RR (95% CI)	*p*
**Codominant inheritance model**					
*BCL2*–CC	57	37	1.00 (ref.)	1 (ref.)	
*BCL2*–CA	42	58	2.12 (1.19–3.77)	1.44 (1.08–1.91)	0.009
*BCL2*–AA	06	05	1.28 (0.36–4.51)	1.11 (0.63–1.95)	0.069
**Dominant inheritance model**					
*BCL2*–CC	57	37	1.00 (ref.)	1 (ref.)	
*BCL2* (CA + AA)	48	63	2.02 (1.15–3.53)	1.40 (1.07–1.83)	0.013
**Recessive inheritance model**					
*BCL2* (CC + CA)	99	95	1.00 (ref.)	1 (ref.)	
*BCL3*–AA	06	05	0.88 (0.25–2.94)	0.93 (0.53–1.63)	0.82
**Allele**					
*BCL2*–C	156	132	1.00 (ref.)	1 (ref.)	
*BCL2*–A	54	68	1.48 (0.97–2.28)	1.22 (0.97–1.53)	0.06
**Overdominant inheritance model**					
*BCL2*-CA	42	58	1.00 (ref.)	1 (ref.)	
*BCL2*-CC + AA	63	42	0.48 (0.28–0.84)	0.70 (0.53–0.92)	0.012

Abbreviations: OR, odds ratio; RR, relative risk ratio.

**Table 5:** Association of *MCL1*-*rs9803935 T >* G gene variation in cases and controls.

Genotypes	Controls *n* = 105	Cases *n* = 100	OR (95% CI)	RR (95% CI)	*p*
**Codominant inheritance model**					
*MCL1*–TT	58	36	1.00 (ref.)	1.00 (ref.)	
*MCL1*–TG	45	54	1.93 (1.08–3.43)	1.35 (1.03–1.77)	0.024
*MCL1*–GG	07	10	2.30 (0.80–6.58)	1.49 (0.83–2.70)	0.120
**Dominant inheritance model**					
*MCL1*–TT	58	36	1.00 (ref.)	1.00 (ref.)	
*MCL1*– (TG + GG)	52	64	1.98 (1.13–3.45)	1.37 (1.06–1.78)	0.015
**Recessive inheritance model**					
*MCL1*-(*TT + TG*)	103	90	1.00 (ref.)	1.00 (ref.)	
*MCL1*–GG	07	10	1.63 (0.59–4.47)	1.29 (0.72–2.32)	0.33
**Allele**					
*MCL1*–T	161	126	1.00 (ref.)	1.00 (ref.)	
*MCL1*–G	57	74	1.65 (1.09–2.51)	1.28 (1.03–1.61)	0.017
**Overdominant inheritance model**					
*MCL1*-TG	45	54	1.00 (ref.)	1.00 (ref.)	
*MCL1-*TT + GG	65	46	0.58 (0.34–1.01)	0.77 (0.59–1.01)	0.058

Abbreviations: OR, odds ratio; RR, relative risk ratio.

**Table 6:** Association of *BIRC5-rs 17882312 C > G* gene variation in cases and controls.

Genotypes	Controls *n* = 105	Cases *n* = 100	OR (95% CI)	RR (95% CI)	*p*
**Codominant inheritance model**					
*BIRC5*-CC	64	31	1.00 (ref.)	1.00 (ref.)	
*BIRC5*-CG	45	65	2.98 (1.68–5.28)	1.64 (1.26–2.14)	0.002
*BIRC5*-GG	01	04	8.25 (0.88–77.0)	3.36 (0.58–19.5)	0.06
**Dominant inheritance model**					
*BIRC5*–CC	64	31	1.00 (ref.)	1.00 (ref.)	
*BIRC5*-(CG + GG)	46	69	3.09 (1. 75–5.46)	1.68 (1.29–2.19)	0.001
**Recessive inheritance model**					
*BIRC5-*(CC + CG)	109	96	1.00 (ref.)	1.00 (ref.)	
*BIRC5*-GG	01	04	4.54 (0.49–41.3)	2.65 (0.45–15.4)	0.179
**Allele**					
*BIRC5*-C	173	127	1.00 (ref.)	1.00 (ref.)	
*BIRC5*-G	47	73	2.11 (1.37–3.25)	1.47 (1.15–1.87)	0.007
**Overdominant inheritance model**					
*BIRC5*-GC	45	65	1.00 (ref.)	1.00 (ref.)	
*BIRC5*-GG + CC	65	35	0.37 (0.21–0.65)	0.62 (0.48–0.82)	0.006

Abbreviations: OR, odds ratio; RR, relative risk ratio.

**Table 7:** Association of *BIRC5-rs 9904341 G > C* gene variation in cases and controls.

Genotypes	Controls *n* = 105	Cases *n* = 100	OR (95% CI)	RR (95% CI)	*p*
**Codominant inheritance model**					
*BIRC5-*GG	70	29	1.00 (ref.)	1.00 (ref.)	
*BIRC5-*GC	50	66	3.18 (1.80–5.62)	1.64 (1.28–2.09)	0.001
*BIRC5-*CC	02	05	6.0 (1.10–32.9)	2.47 (0.76–8.03)	0.037
**Dominant inheritance model**					
*BIRC5-*GG	70	29	1.00 (ref.)	1.00 (ref.)	
*BIRC5-*(GC + CC)	52	71	3.29 (1.87–5.77)	1.67 (1.31–2.13)	0.001
**Recessive inheritance model**					
*BIRC5-*(GG + GC)	120	95	1.00 (ref.)	1.00 (ref.)	
*BIRC5-*CC	02	05	3.15 (0.59–16.6)	1.95 (0.60–6.34)	0.17
**Allele**					
*BIRC5-*G	190	124	1.00 (ref.)	1.00 (ref.)	
*BIRC5-*C	57	76	2.04 (1.35–3.08)	1.30 (1.03–1.63)	0.001
Overdominant inheritance model					
*BIRC5-*GC	50	66	1.00 (ref.)	1.00 (ref.)	
*BIRC5-*GG + CC	72	34	0.35 (0.20–0.61)	0.88 (0.64–1.23)	0.002

Abbreviations: OR, odds ratio; RR, relative risk ratio.

## Data Availability

The data presented in this study are available on request from the corresponding author.

## Supplementary Materials

The following supporting information can be downloaded at: https://www.mdpi.com/article/10.3390/cancers15041012/s1, Table S1. Protocol. Table S2. ARMS primers for the *BLC2, MCL1*, and *Survivin* genes mutation. Table S3. Whole exome sequencing of *BCL2* gene in lymphoma cases.
