# Supplementary material for: Antiapoptotic Gene Genotype and Allele Variations and the Risk of Lymphoma
Source: Cancers (Basel). 2023 Feb 5;15(4):1012. doi: 10.3390/cancers15041012 (PMC9954290; doi:10.3390/cancers15041012)
Supplement: Supplementary file 1 [file cancers-15-01012-s001.zip › Table S1.pdf]

Table S1. Protocol

Sample quality control (QC)	Library Prep Protocol	Sequencing Protocol
The DNA samples were measured using the Qubit DNA Assay BR (Invitrogen, Cat# Q32853). DNA integrity was examined on a 1% Agarose gel, and DNA purity was verified using QIAxpert (Agilent, Cat. No. 5067-5365). All 25 QC-passed samples were taken for library creation and sequencing after confirmation.	Whole genome libraries were prepared using the Twist Library Preparation EF Kit (Twistbio, Cat# 101058) methodology and then captured utilizing a unique gene panel (Leukemia panel). In a series of enzymatic processes, 50 ng of DNA were enzymatically fragmented to roughly 250 bp, end-repaired, A-tailed, and adapter-ligated. Following adapter ligation, the products were purified using Agencourt® AMPure® XP beads (Beckman Coulter, Cat# A63882) and PCR enriched under the following thermal conditions: initial denaturation at 98°C for 45 sec; 7 cycles at 98°C for 15 sec; 60°C for 30 sec; 72°C for 30 sec; and final extension at 72°C for 1 min. Twist Custom Probes (LeukemiaHG38) were used to capture specific regions of the generated genome libraries. The library fragments were hybridized into the biotinylated probes. These beads were then captured on streptavidin beads. Using D1000 DNA ScreenTapes (Agilent, Cat# 5067-5582) or a Fragment Analyzer with a dsDNA Reagent Kit (1-6000 bp) (Agilent, Cat# 5191-6576), the final exome collected libraries were examined for fragment size distribution.	With the help of the Qubit High Sensitivity Assay (Invitrogen, Cat# Q32851), prepared libraries were quantified. The resultant libraries were combined and diluted to get the ideal loading concentration. The Illumina Novaseq V1.5 instrument was then loaded with the pooled libraries to produce 2X, 150 bp paired-end reads for each sample.
