# Supplementary material for: Antiapoptotic Gene Genotype and Allele Variations and the Risk of Lymphoma
Source: Cancers (Basel). 2023 Feb 5;15(4):1012. doi: 10.3390/cancers15041012 (PMC9954290; doi:10.3390/cancers15041012)
Supplement: Supplementary file 1 [file cancers-15-01012-s001.zip › Table S3.pdf]

Table S3. Whole exome sequencing of *BCL2* gene in lymphoma cases

#	Variant	c-DNA_CHG	AA_CHG
1.	FRAMESHIFT-DEL	c.245del	p. Ala82GlyfsTer14
2.	MISSENSE	c.239C>G	p. Ala80Gly
3.	FRAMESHIFT-DEL	c.234del	p. Gly79AlafsTer17
4.	FRAMESHIFT-INS	c.233_234insG	p. Gly79ArgfsTer74
5.	FRAMESHIFT-DEL	c.511del	p. Asp171ThrfsTer7
6.	MISSENSE	c.149C>T	p. Ser50Phe
7.	FRAMESHIFT-DEL	c.124del	p. Ala42ProfsTer54
8.	FRAMESHIFT-DEL	c.110_113del	p. Ala37GlyfsTer58
9.	MISSENSE	c.520G>T	p. Ala174Ser
10.	MISSENSE	c.167C>T	p. Thr56Met
11.	FRAMESHIFT-DEL	c.698del	p. Gly233ValfsTer25
12.	FRAMESHIFT-DEL	c.119del	p. Pro40ArgfsTer56
13.	MISSENSE	c.347C>T	p. Ser116Phe
14.	SILENT	c.231C>T	p. Ala77(=)
15.	MISSENSE	c.125C>T	p. Ala42Val
16.	FRAMESHIFT-DEL	c.508del	p. Val170TrpfsTer8
17.	FRAMESHIFT-DEL	c.126del	p. Ala43ProfsTer53
18.	MISSENSE	c.91G>A	p. Asp31Asn
19.	FRAMESHIFT-DEL	c.612del	p. Ser205AlafsTer13
20.	MISSENSE	c.298G>A	p. Ala100Thr
21.	MISSENSE	c.250C>T	p. Pro84Ser
22.	MISSENSE	c.537G>C	p. Glu179Asp
23.	MISSENSE	c.338C>G	p. Ala113Gly
24.	MISSENSE	c.308A>G	p. Asp103Gly
25.	MISSENSE	c.256C>G	p. Leu86Val
26.	MISSENSE	c.215T>C	p. Leu72Pro
27.	MISSENSE	c.214C>A	p. Leu72Met
28.	MISSENSE	c.184T>C	p. Ser62Pro
29.	MISSENSE	c.175C>T	p. Pro59Ser
30.	MISSENSE	c.40A>C	p. Ile14Leu
31.	MISSENSE	c.39G>C	p. Glu13Asp
32.	MISSENSE	c.23G>A	p. Gly8Glu
33.	MISSENSE	c.20C>G	p. Thr7Arg
34.	MISSENSE	c.581G>A	p. Gly194Asp
35.	FRAMESHIFT-DEL	c.466del	p. Val156SerfsTer22
36.	FRAMESHIFT-DEL	c.352del	p. Gln118SerfsTer4
37.	MISSENSE	c.185C>T	p. Ser62Phe
38.	MISSENSE	c.179C>T	p. Ala60Val
39.	FRAMESHIFT-DEL	c.172del	p. His58IlefsTer38
40.	MISSENSE	c.156G>T	p. Gln52His

41.	1.	FRAMESHIFT-INS	c.119dup	p. Ala42GlyfsTer111
42.	2.	MISSENSE	c.70T>G	p. Ser24Ala
43.	3.	MISSENSE	c.17G>C	p. Arg6Thr
44.	4.	MISSENSE	c.461G>A	p. Gly154Asp
45.	5.	FRAMESHIFT-INS	c.125_126insGCGG	p. Ala43ArgfsTer111
46.	6.	MISSENSE	c.434G>A	p. Gly145Glu
47.	7.	MISSENSE	c.325C>T	p. Arg109Cys
48.	8.	MISSENSE	c.206C>T	p. Thr69Ile
49.	9.	MISSENSE	c.157C>T	p. Pro53Ser
